# Prophylactic temporary abdominal aortic balloon occlusion for patients with pernicious placenta previa: a retrospective study

**DOI:** 10.1186/s12871-021-01354-1

**Published:** 2021-04-29

**Authors:** Fei Huo, Hansheng Liang, Yi Feng

**Affiliations:** Department of Anesthesiology, Peking University People’s Hospital, Beijing, 100044 China

**Keywords:** Prophylactic abdominal aorta balloon occlusion, Pernicious placenta previa, Caesarean section

## Abstract

**Background:**

Pernicious placenta previa (PPP) can increase the risk of perioperative complications. During caesarean section in patients with adherent placenta, intraoperative blood loss, hysterectomy rate and transfusion could be reduced by interventional methods. Our study aimed to investigate the influence of maternal hemodynamics control and neonatal outcomes of prophylactic temporary abdominal aortic balloon (PTAAB) occlusion for patients with pernicious placenta previa.

**Methods:**

This was a retrospective study using data from the Peking University People’s Hospital from January 2014 through January 2020. Clinical records of pregnant women undergoing cesarean section were collected. Patients were divided into two groups: treatment with PTAAB placement (group A) and no balloon placement (group B). Group A was further broken down into two groups: prophylactic placement (Group C) and balloon occlusion (group D).

**Results:**

Clinical records of 33 cases from 5205 pregnant women underwent cesarean section were collected. The number of groups A, B, C, and D were 17, 16, 5 and 12.We found that a significant difference in the post-operative uterine artery embolism rates between group A and group B (0% vs.31.3%, *p* = 0.018). There was a significant difference in the Apgar scores at first minute between group A and group B (8.94 ± 1.43 vs 9.81 ± 0.75,*p* = 0.037),and the same significant difference between two groups in the pre-operative central placenta previa (29.4% vs. 0%,*p* = 0.044), complete placenta previa (58.8% vs 18.8%, *p* = 0.032),placenta implantation (76.5% vs 31.3%, *p* = 0.015). We could also observe the significant difference in the amount of blood cell (2.80 ± 2.68vs.10.66 ± 11.97, *p* = 0.038) and blood plasma transfusion (280.00 ± 268.32 vs. 1033.33 ± 1098.20, *p* = 0.044) between group C and group D. The significant differences in the preoperative vaginal bleeding conditions (0% vs 75%, *p* = 0.009), the intraoperative application rates of vasopressors (0% vs. 58.3%, *p* = 0.044) and the postoperative ICU (intensive care unit) admission rates (0% vs. 58.3%, *p* = 0.044) were also kept.

**Conclusions:**

PTAAB occlusion could be useful in reducing the rate of post-operative uterine artery embolism and the amount of transfusion, and be useful in coping with patients with preoperative vaginal bleeding conditions, so as to reduce the rate of intraoperative applications of vasopressors and the postoperative ICU (intensive care unit) admission. In PPP patients with placenta implantation, central placenta previa and complete placenta previa, we advocate the utilization of prophylactic temporary abdominal aortic balloon placement.

## Background

Placenta previa refers to the placenta partially or completely blocking the lower uterine cervix of the endometrial orifice [1]. Over these years, the rate of morbidly adherent placenta (MAP) has increased, exceeding to 1 in 2500 in the 1980s and 1990s. Recent data suggest that this trend continues to rise [2]. Recent studies have shown that placenta previa, repetitive cesarean section (CS) and uterine abnormalities are hazard factors for placenta previa in the general population [3].

Placenta previa has serious adverse clinical consequences for mothers and infants, prenatal and intrapartum hemorrhage, premature delivery and emergency hysterectomy [4]. Pernicious placenta previa refers to a pregnant patient with a history of caesarean section with placenta previa and a high risk of placental accrete. Pernicious placenta previa is characterized as placenta previa that adheres to previous cesarean scars [5].

Therefore, exploring effective methods is necessary to reduce massive blood loss during cesarean delivery and reserve the uterine function of women who are with pernicious placenta previa.

We’ve known that in pregnant patients with pernicious placenta previa who are undergoing CS, prophylactic lower abdominal aorta balloon occlusion, internal iliac balloon occlusion, uterine artery occlusion and other kinds of intervention methods can be considered as effective methods to reduce intraoperative blood loss, transfusion and hysterectomy rate [5–13]. Previous study has shown that the amount of intraoperative blood loss can be well controlled by the combination of the abdominal aorta balloon occlusion on the lower uterine segment [14]. But no institute previously had ever reported the cases with prophylactic balloon placement,but without balloon occlusion.

In our institute, some cases with the prophylactic aortic balloon placement hadn’t been occluded during the surgery. Hence, we conducted a retrospective study and aimed to compare the different characteristics of pernicious placenta previa with and without placement or occlusion of prophylactic abdominal aortic balloon.

## Methods

The study protocol was approved by the Ethics Committee of Peking University People’s Hospital (2020PHB184–01). We identified women admitted to our hospital between January 2014 through January 2020 with prenatally diagnosed pernicious placenta previa by searching our electronic medical record database with the following keywords: pernicious placenta previa. The inclusion criteria were as follows:(1) pernicious placenta previa diagnosed by color Doppler ultrasonography or magnetic resonance imaging (MRI), a history of at least one previous CS, and patients without other obstetric diseases;(2) completed information of patients’ features were available. Patients with missing clinical data and those complicated with other obstetric diseases were excluded from our study.

The information was recorded from the electronic anesthetic documentation system.

The demographic data was collected including age, BMI (body mass index), pregnancy week, pregnancy times, parity times, prior cesarean section times and the overall balloon occlusion time. The clinical data included estimated blood loss, total transfusion, red blood cell transfusion, blood plasma transfusion, platelet transfusion, fibrinogen transfusion, prothrombin complex transfusion, preoperative hemoglobin, preoperative creatine, preoperative urea nitrogen, postoperative hemoglobin at 24 h, postoperative creatine at 24 h, postoperative urea nitrogen at 24 h, modes of anesthesia (spinal anesthesia, non-spinal anesthesia), preoperative ureteral stent placement and postoperative hospital stay. Data regarding maternal hemodynamics control which included the intraoperative application of vasopressor, hysterectomy, postoperative uterine artery embolism, postoperative ICU (intensive care unit) admission was collected. Data regarding neonatal outcome which included neonatal weight, APGAR score at 1 min, 5 min and 10 min was collected.

About grouping, according to the surgeon’s comprehensive preoperative considerations and the patient’s willingness, aortic balloon insertions were performed in some of the patients with pernicious placenta previa. Patients were divided into two groups: treatment with PTAAB placement (group A) and no balloon placement (group B).

The procedure for the management of abdominal aortic balloon was as follows. On the day of surgery, the woman was first transferred to the interventional operating room for insertion of the aortic balloons under fluoroscopic guidance. The procedure was performed by vascular surgeons. Catheters were inserted bilaterally via the femoral arteries under local anesthesia, and the tip placed at the infrarenal aortic artery. The balloon was briefly inflated, and contrast was injected to verify occlusion of the artery. After placement of the catheters, the woman was transferred to the obstetric operating room. The peri-operative management was conducted by the anesthetists. According to the process of the operation, some balloons hadn’t been inflated mainly because the blood loss was well controlled by surgical hemostasis. But for those who experienced rapid blood loss and fatal fluctuation of circulation, the balloons had been inflated. Group A was further broken down into two groups: prophylactic placement (Group C) and balloon occlusion (group D).

Primary outcomes included estimated intraoperative blood loss, amount of intra-operative blood transfusion, rate of the application of vasopressor, the admission to ICU (intensive care unit), rate of uterine artery embolization and hysterectomy. Blood loss was determined by weighing surgical sponges and measuring suction drainage. Data regarding maternal hemodynamics control and neonatal outcome was collected.

### Statistical analysis

Statistical significance was calculated using the Chi-square or Fisher exact tests for differences in qualitative variables and the independent sample t test for differences in continuous variables, and a value of *P* < 0.05 was considered significant. The statistical package SPSS for Windows, release 21.0 was used for data analysis.

## Results

**Fig. 1:**
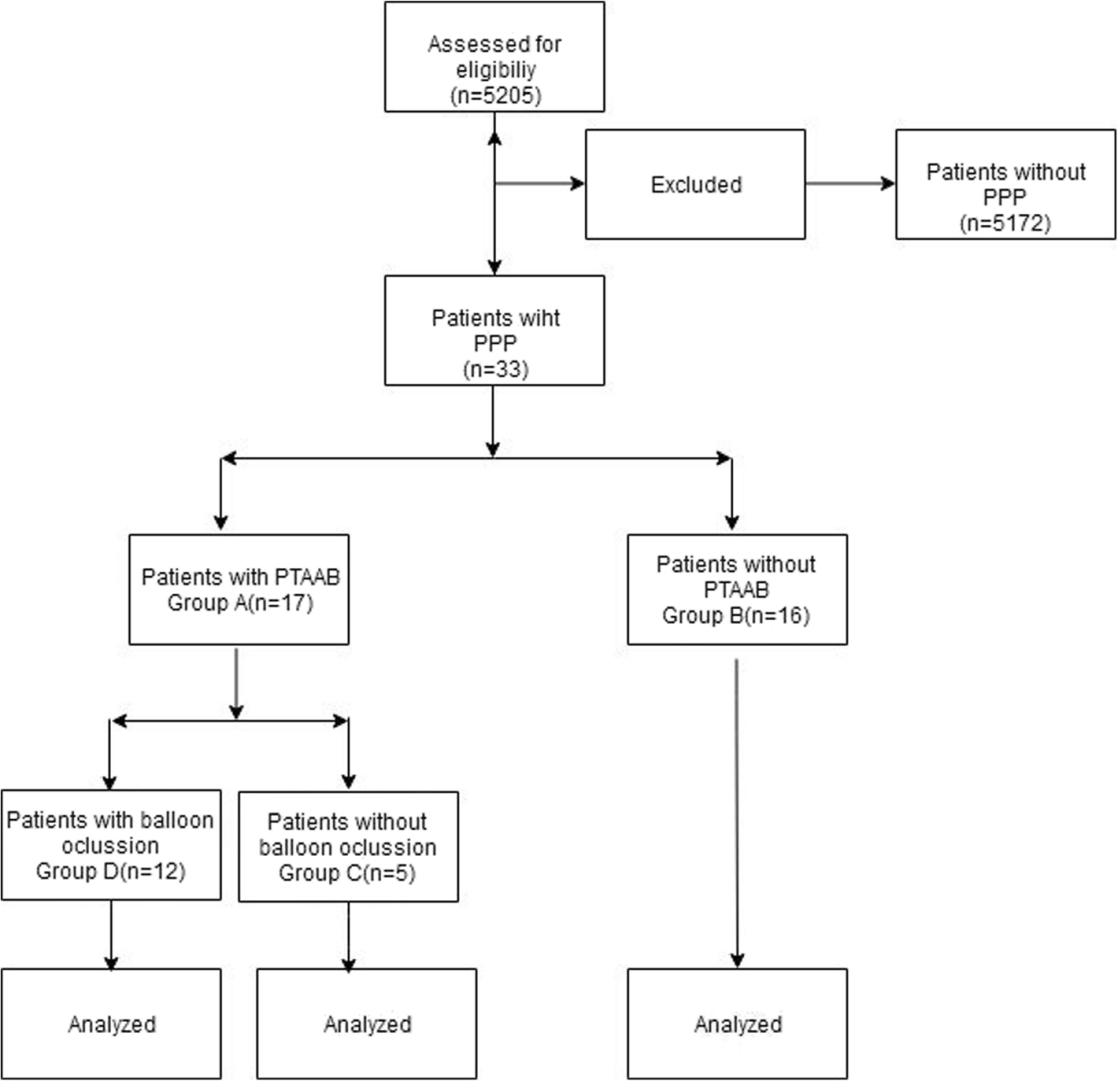
Study Flow

As listed in Fig. 1, a total of 5205 pregnant women underwent cesarean section between January 2014 through January 2020 in our hospital. After excluding cases whose preoperative diagnosis was not PPP, we included 33 pregnant women meeting the inclusion criteria.

The rate of utilization of the technique of PTAAB was 51.5% (17/33) among the patients diagnosed with pernicious placenta previa. The rate of balloon occlusion in PTAAB patients was 70.6%(12/17). And the average overall balloon occlusion time was 39.70 min (Table 1). Patients were divided into PTAAB utilization group (Group A) and non-PTAAB utilization group (Group B) based on the application of PTAAB. Group A was further broken down into patients who required balloon occlusion (group D) and those that did not, despite prophylactic placement (Group C).

**Table 1:** Demographic characteristics of patients with PPP (*n* = 33)

‘	GroupA(***n*** = 17)	GroupB(***n*** = 16)	***P*** value
Age (years old)	32.82 ± 4.45	34.44 ± 4.79	0.323
BMI (kg/m2)	27.53 ± 2.69	27.60 ± 3.49	0.950
Pregnancy length (week)	36.82 ± 1.98	35.44 ± 2.34	0.075
Pregnancy (number)	3.24 ± 1.20	3.81 ± 1.24	0.217
Parity (number)	1.65 ± 0.49	1.94 ± 0.85	0.237
Prior Cesarean section (number)	1.06 ± 0.24	1.25 ± 0.58	0.234
Overall balloon occlusion time (min)	39.70 ± 58.08	–	–
Complete placenta previa	10 (58.8%)	3 (18.8%)	0.032*
Central placenta previa	5 (29.4%)	0	0.044*
Placenta implantation	13 (76.5%)	5 (31.3%)	0.015*
Preoperative vaginal bleeding	9 (52.9%)	8 (50%)	1.000

*PPP* pernicious placenta previa

*BMI* body mass index

*p-value less than 0.05 was considered statistically significant

Data regarding maternal hemodynamics control and neonatal outcome were also collected.

There was a significant difference in the Apgar scores at first minute between group A and group B (8.94 ± 1.43 vs 9.81 ± 0.75,*p* = 0.037),and the same significant difference between two groups in the pre-operative central placenta previa (29.4% vs. 0%,*p* = 0.044), complete placenta previa (58.8% vs 18.8%, *p* = 0.032),placenta implantation (76.5% vs 31.3%, *p* = 0.015) (Table 2).

To further determine the effect of balloon occlusion on maternal and neonatal outcomes, patients in Group A were stratified based on whether they received balloon occlusion (Group D,*n* = 12) or not (Group C,*n* = 5) (Table 3). We could also observe the significant difference in the amount of blood cell (2.80 ± 2.68 vs.10.66 ± 11.97, *p* = 0.038) and blood plasma transfusion (280.00 ± 268.32 vs. 1033.33 ± 1098.20, *p* = 0.044) between group C and group D (Table 4). The significant differences in the preoperative vaginal bleeding conditions (0% vs 75%, *p* = 0.009), the intraoperative application rates of vasopressors (0% vs. 58.3%, *p* = 0.044) and the postoperative ICU (intensive care unit) admission rates (0% vs. 58.3%, *p* = 0.044) were also kept. The reduction in vasopressor use and ICU stay was specific to group D (Table 5).

**Table 2:** Maternal hemodynamics control and neonatal outcome in patients with PPP (*n* = 33)

‘	GroupA (***n*** = 17)	GroupB (***n*** = 16)	***P*** value
Intraoperative application of vasopressor	7 (41.2%)	12 (75%)	0.080
Hysterectomy	3 (17.6%)	2 (12.5%)	1.000
Postoperative uterine artery embolism	0	5 (31.3%)	0.018*
Postoperative ICU admission	7 (41.2%)	8 (50%)	0.494
Neonatal weight(g)	2638.24 ± 346.07	2580.31 ± 503.58	0.071
APGAR 1MIN(score)	8.94 ± 1.43	9.81 ± 0.75	0.037^#^
APGAR 5MIN(score)	9.82 ± 0.39	9.94 ± 0.25	0.326
APGAR10MIN(score)	9.88 ± 0.33	10.00 ± 0.00	0.163

*PPP* pernicious placenta previa

^#^*p*-value less than 0.05 was considered statistically significant

**Table 3:** Demographic characteristics of patients with PTAAB (*n* = 17)

	GroupC (***n*** = 5)	GroupD (***n*** = 12)	***P*** value
Age (years old)	34.60 ± 5.17	32.08 ± 4.12	0.303
BMI (kg/m2)	27.78 ± 2.15	27.42 ± 2.96	0.808
Pregnancy length (week)	37.80 ± 2.04	36.42 ± 1.88	0.198
Pregnancy (number)	3.80 ± 2.16	3.00 ± 0.42	0.458
Parity (number)	1.80 ± 0.44	1.58 ± 0.51	0.237
Prior Cesarean section (number)	1.00 ± 0.00	1.08 ± 0.28	0.536
Complete placenta previa	3 (60%)	7 (58.3%)	1.000
Central placenta previa	2 (40%)	3 (25%)	0.600
Placenta implantation	3 (60%)	10 (83.3%)	0.538
Preoperative vaginal bleeding	0	9 (75%)	0.009*

*BMI* body mass index

PTAAB prophylactic temporary abdominal aortic balloon

*p-value less than 0.05 was considered statistically significant

**Table 4:** Clinical characteristics of patients with PTAAB (*n* = 17)

	GroupC (***n*** = 5)	GroupD (***n*** = 12)	***P*** value
Estimated blood loss (ml)	1740.00 ± 750.33	3762.50 ± 3728.33	0.096
Total transfusion (ml)	3130.00 ± 1159.52	6484.33 ± 4711.29	0.143
Red blood cell transfusion(u)	2.80 ± 2.68	10.66 ± 11.97	0.038#
Blood plasma transfusion (ml)	280.00 ± 268.32	1033.33 ± 1098.20	0.044#
Platelet transfusion(u)	0.00 ± 0.00	0.58 ± 1.16	0.289
Fibrinogen transfusion(g)	0.00 ± .0.00	2.04 ± 3.22	0.082
Prothrombin complex transfusion(u/100)	0.00 ± .0.00	4.00 ± 7.23	0.802
Postoperative hospital stay (day)	6.80 ± 2.95	8.25 ± 5.37	0.583
Preoperative hemoglobin(g/L)	116.80 ± 14.75	112.50 ± 13.77	0.594
Preoperative creatine (umol/L)	47.80 ± 3.19	53.08 ± 14.94	0.454
Preoperative urea nitrogen (mmol/L)	3.57 ± 1.33	3.36 ± 0.97	0.726
Postoperative hemoglobin at 24 h(g/L)	100.00 ± 25.52	99.17 ± 16.19	0.932
Postoperative creatine at 24 h (umol/L)	58.60 ± 24.37	59.25 ± 25.01	0.961
Postoperative urea nitrogen at 24 h (mmol/L)	3.07 ± 1.22	3.16 ± 1.54	0.912
Spinal anesthesia	2 (11.8%)	6 (50%)	0.080
Preoperative ureteral stent placement	5 (100%)	7 (58.3%)	0.245

*PTAAB* prophylactic temporary abdominal aortic balloon

#*p*-value less than 0.05 was considered statistically significant

**Table 5:** Maternal hemodynamics control and neonatal outcome with PTAAB (*n* = 17)

	GroupC (***n*** = 5)	GroupD (***n*** = 12)	***P*** value
Intraoperative application of vasopressor	0	7 (58.3%)	0.044*
Hysterectomy	0	3 (25%)	0.515
Postoperative uterine artery embolism	0	0	1.00
Postoperative ICU admission	0	7 (58.3%)	0.044*
Neonatal weight(g)	2616.00 ± 361.49	2647.50 ± 355.48	0.871
APGAR 1MIN(score)	9.20 ± 1.30	8.83 ± 1.52	0.646
APGAR 5MIN(score)	10.00 ± 0.00	9.75 ± 0.45	0.082
APGAR10MIN(score)	10.00 ± 0.00	9.83 ± 0.38	0.362

*PTAAB* prophylactic temporary abdominal aortic balloon

*p-value less than 0.05 was considered statistically significant

## Discussion

For patients with placenta previa, preoperative prophylactic balloon occlusion can reduce cesarean hysterectomy [15]. The location of the balloon catheter remains debatable. The choice of the site of aortic occlusion may depend on the individual. The damage caused by occlusion and the compensation of collateral circulation could come to a balance in these patients [16, 17]. Angiography of collateral circulation from the ligamentum teres artery to the uterus during cesarean section could be a risk factor for massive blood loss in patients with BOIA (balloon occlusion of the internal iliac artery) [18].

Due to the insufficiency of the occlusion of IIA (internal iliac artery) and CIA (common iliac artery), balloon catheterization of the aorta could have better clinical results. In a previous study, in patients with PPP accompanied by infrarenal abdominal aorta balloon occlusion, the estimated blood loss (ml) was 1600.00 ± 1185.785, the hysterectomy rate was 8.3%, and the ICU admission rate was 37.5% [19, 20]. In our study, the estimated blood loss (ml) was 3167.65 ± 3255.71, the hysterectomy rate was 17.6%, and the ICU admission rate was 41.2% (Table 6).

**Table 6:** Clinical characteristics of patients with PPP (*n* = 33)

‘	GroupA (***n*** = 17)	GroupB (***n*** = 16)	***P*** value
Estimated blood loss (ml)	3167.65 ± 3255.71	2831.25 ± 1906.03	0.722
Tota transfusion (ml)	5497.76 ± 4251.83	5200.00 ± 2580.18	0.811
Red blood cell transfusion(u)	8.35 ± 10.01	8.00 ± 5.84	0.903
Blood plasma transfusion (ml)	811.76 ± 986.08	712.50 ± 565.54	0.727
Platelet transfusion(u)	0.41 ± 1.00	0.25 ± 0.45	0.559
Fibrinogen transfusion(g)	1.44 ± 2.84	0.94 ± 1.39	0.520
Prothrombin complex transfusion(u/100)	2.82 ± 6.29	3.38 ± 6.23	0.802
Postoperative hospital stay (day)	7.82 ± 4.75	6.75 ± 2.57	0.429
Preoperative hemoglobin(g/L)	113.76 ± 13.75	110.00 ± 21.49	0.551
Preoperative creatine (umol/L)	51.53 ± 12.74	42.69 ± 9.04	0.029#
Preoperative urea nitrogen (mmol/L)	3.43 ± 1.05	2.82 ± 0.94	0.089
Postoperative hemoglobin at 24 h(g/L)	99.41 ± 17.53	95.00 ± 13.07	0.421
Postoperative creatine at 24 h (umol/L)	59.06 ± 24.06	45.19 ± 11.20	0.044^#^
Postoperative urea nitrogen at 24 h (mmol/L)	3.13 ± 1.42	2.69 ± 1.03	0.315
Spinal anesthesia	8 (47.1%)	9 (56.3%)	0.732
Preoperative ureteral stent placement	12 (70.6%)	10 (62.5%)	0.721

*PPP* pernicious placenta previa

^#^*p*-value less than 0.05 was considered statistically significant

In addition, Uterine artery embolization (UAE) or OAE (ovarian artery embolization) after prophylactic abdominal aortic balloon occlusion can effectively control postpartum hemorrhage, reduce blood loss, blood transfusion and hysterectomy rates. In a previous study, cesarean section was conducted with the occlusion balloon technique followed by uterine or ovarian artery embolization. Nine cases of bleeding happened after the release of the balloon. The bleeding originated from the ovarian arteries and uterine arteries. And doctors performed further embolization. The uterus conserving rate was 96.77% [7].

Transcatheter uterine artery embolization is not enough for decreasing postpartum hemorrhaging due to incomplete embolization of the blood supply of the uterus. Abdominal aortic occlusion has many benefits [21]. As a consequence, the embolism rate of uterus was decreased in group A in our study. We find that prophylactic temporary abdominal aortic balloon occlusion could be useful reducing the rate of post-operative uterine artery embolism.

When it comes to the neonatal outcome,in one previous case, the Apgar scores were three at 1 min and seven at 5 min and the umbilical cord venous pH was 6.95.The low umbilical Apgar scores could be due to decreased uterine perfusion from the disruption of the iliac artery [22].. Another previous study has indicated that the five-minute Apgar score was a better predictor of neonatal outcome than the umbilical-artery blood pH, even for newborn infants with severe acidemia [23]. In our study, Apgar score at 1 min in Group A (8.94 ± 1.43) was lower than in Group B (9.81 ± 0.75). But Apgar scores at 5 min didn’t differ between these two groups. In this spective, the neonatal outcome didn’t seem to be interrupted by PTAAB. But it still raises our attention for neonatal outcomes in PPP patients with PTAAB.

In our study, the prophylactic temporary abdominal aortic balloon was placed. We find that prophylactic temporary abdominal aortic balloon occlusion could be useful in coping with pernicious placenta previa with placenta implantation, central placenta previa, complete placenta previa.

In addition, we analyze the 17 patients with balloon placement. Not all the balloons are inflated during the surgery. We want to explore the effect of balloon occlusion on maternal and neonatal outcomes. To cope with massive blood loss, there is need for fluid resuscitation, blood products transfusion and the application of vasopressors.

In our retrospective study, the incidence of preoperative vaginal bleeding between group C and D is different. Vaginal bleeding is possibly to happen when the lower segment of the uterus comes to form from 32 weeks of pregnancy in patients with placenta previa. Severe bleeding in placenta previa is associated with high risk of maternal morbidity [24]. And the rate of intraoperative applications of vasopressors, the amount of intraoperative blood cell and plasms transfusion and the postoperative ICU (intensive care unit) admission appear different in balloon occlusion (Group D) and non balloon occlusion (Group C).

Upon the anesthetic method, there exists no difference in the ratio of spinal anesthesia in these groups*(p* > 0.05). In one study, the reporter preferred for neuraxial anesthesia in the absence of contraindications during abdominal aorta balloon catheterization intervention when treating patients diagnosed as placenta previa and suspicion for placenta accrete [25]. Since the pathophysiology varies along with the process of the operation, some cases are conducted with the combination of spinal anesthesia and general anesthesia due to massive blood loss.

Not only do we care about the anesthetic method, we also care about the perioperative renal functions. Previous study has shown that perioperative placement of internal iliac artery occlusion balloon is safe [26]. In our study, the postoperative creatine levels appear significant different in group A and group B*(P* < 0.05). So we need to be more cautious about the perioperative renal functions of the patients and find the balance between blood loss control and preserving renal functions. In order not to disturb the blood flow of renal artery, the balloons are placed infrarenal in our study. But there could exist a bias, because the preoperative creatinine in group A is higher and group A seems to have more risks of renal disruption. The post-creatine levels were increased in both groups, which might suggest the potential renal injury might not result from balloon, but from intra-operative management, such as persistent hypotension. Also, although there could exist difference in group A and group B, the creatine level in these two groups are within the normal level.

However, renal perfusion was reduced regardless of the location of the aortic obstruction. At the same time, the patients had adverse consequences of ischemia-reperfusion. One previous study indicated that the Aortic Occlusion for Resuscitation in Trauma and Acute Care Surgery (AORTA) registry showed a low rate of acute kidney injury. Nevertheless, drug therapy to reduce reperfusion injury appears to be disappointing. Improving the outcomes of patients with PPP requires multiple-approach managements, of which, cell salvage is an important one [27]. The best recommendation for the anesthesiologist is to optimize hemodynamic status and adjust the circulating blood volume for favorable renal perfusion [28].

### Limitations

The main limitation of our study is its retrospective characteristics. Because subjects were not randomly allocated, selection bias may exist. The number of cases is also too small in our study, and this study could not be adequately powered statistically. And the medical intervention (such as the choice of patients to receive balloon or not, the determination to inflate the balloon or not.) cannot be made on a blinded base. As a result, a large multicenter and randomized controlled study is needed to verify the findings.

## Conclusion

Prophylactic temporary abdominal aortic balloon occlusion could be useful in reducing the rate of post-operative uterine artery embolism and the amount of transfusion. Prophylactic temporary abdominal aortic balloon occlusion could also be useful in coping with patients with preoperative vaginal bleeding conditions, and reducing the rate of intraoperative applications of vasopressors and the postoperative ICU (intensive care unit) admission. In PPP patients with placenta implantation, central placenta previa and complete placenta previa, we advocate the utilization of prophylactic temporary abdominal aortic balloon placement.

## Data Availability

The datasets used and/or analyzed during the current study are available from the corresponding author on reasonable request.

## Abbreviations

PPP: Pernicious placenta previa
PTAAB: Prophylactic temporary abdominal aortic balloon
ICU: Intensive care unit
MAP: Morbidly adherent placenta
CS: Cesarean sections
MRI: Magnetic resonance imaging
BMI: Body mass index
BOIA: Balloon occlusion of the internal iliac artery
IIA: Internal iliac artery
CIA: Common iliac artery
UAE: Uterine arterial embolization
OAE: Ovarian arterial embolization
EIA: External iliac artery
IMA: Inferior mesenteric artery
AORTA: Aortic Occlusion for Resuscitation in Trauma and Acute Care Surgery
